# Inflammation and Immune Reactions in the Fetus as a Response to COVID-19 in the Mother

**DOI:** 10.3390/jcm12134256

**Published:** 2023-06-25

**Authors:** Nilufar R. Gashimova, Liudmila L. Pankratyeva, Victoria O. Bitsadze, Jamilya Kh. Khizroeva, Maria V. Tretyakova, Kristina N. Grigoreva, Valentina I. Tsibizova, Jean-Christophe Gris, Natalia D. Degtyareva, Fidan E. Yakubova, Alexander D. Makatsariya

**Affiliations:** 1 Sechenov University, 2 bldg. 4, Bolshaya Pirogovskaya Str., 119991 Moscow, Russia; 2 Dmitry Rogachev National Medical Research Center of Pediatric Hematology, Oncology and Immunology, 1 Samory Mashela Street, 117997 Moscow, Russia; 3 Clinical Research Center, Vorokhobov City Clinical Hospital No 67, 2/44 Salama Adil Str., 123423 Moscow, Russia; 4 Federal State Budgetary Institution “Almazov National Medical Research Centre”, Ministry of Health of the Russian Federation 2 Akkuratova Street, 197341 St. Petersburg, Russia; 5 University of Montpellier, 163 Rue Auguste Broussonnet, 34090 Montpellier, France

**Keywords:** COVID-19, fetal inflammatory response syndrome, Treg cells, fetus, pregnancy, cytokines

## Abstract

**Background**: Contracting COVID-19 during pregnancy can harm both the mother and the unborn child. Pregnant women are highly likely to develop respiratory viral infection complications with critical conditions caused by physiological changes in the immune and cardiopulmonary systems. Asymptomatic COVID-19 in pregnant women may be accompanied by fetal inflammatory response syndrome, which has adverse consequences for the newborn’s life and health. **Purpose**: To conduct an inflammatory response assessment of the fetus due to the effects of COVID-19 on the mother during pregnancy by determining pro-inflammatory cytokines, cell markers, T regulatory cells, T cell response, evaluation of cardiac function, and thymus size. **Materials and methods**: A prospective study included pregnant women (*n* = 92). The main group consisted of 62 pregnant women with COVID-19 infection: subgroup 1—SARS-CoV-2 PCR-positive pregnant women 4–6 weeks before delivery (*n* = 30); subgroup 2—SARS-CoV-2 PCR-positive earlier during pregnancy (*n* = 32). The control group consisted of 30 healthy pregnant women. In all pregnant women, the levels of circulating cytokines and chemokines (IL-1α, IL-6, IL-8, IL-10, GM-CSF, TNF-α, IFN-γ, MIP-1β, and CXCL-10) were determined in the peripheral blood and after delivery in the umbilical cord blood, and an analysis was performed of the cell markers on dendritic cells, quantitative and functional characteristics of T regulatory cells, and specific T cell responses. The levels of thyroxine and thyroid-stimulating hormone were determined in the newborns of the studied groups, and ultrasound examinations of the thymus and echocardiography of the heart were also performed. **Results**: The cord blood dendritic cells of newborns born to mothers who suffered from COVID-19 4–6 weeks before delivery (subgroup 1) showed a significant increase in CD80 and CD86 expression compared to the control group (*p* = 0.023). In the umbilical cord blood samples of children whose mothers tested positive for COVID-19 4–6 weeks before delivery (subgroup 1), the CD4+CCR7+ T cells increased with a concomitant decrease in the proportion of naive CD4+ T cells compared with the control group (*p* = 0.016). Significantly higher levels of pro-inflammatory cytokines and chemokines were detected in the newborns of subgroup 1 compared to the control group. In the newborns of subgroup 1, the functional activity of T regulatory cells was suppressed, compared with the newborns of the control group (*p* < 0.001). In all pregnant women with a severe coronavirus infection, a weak T cell response was detected in them as well as in their newborns. In newborns whose mothers suffered a coronavirus infection, a decrease in thymus size, transient hypothyroxinemia, and changes in functional parameters according to echocardiography were revealed compared with the newborns of the control group. **Conclusions**: Fetal inflammatory response syndrome can occur in infants whose mothers suffered from a COVID-19 infection during pregnancy and is characterized by the activation of the fetal immune system and increased production of pro-inflammatory cytokines. The disease severity in a pregnant woman does not correlate with SIRS severity in the neonatal period. It can vary from minimal laboratory parameter changes to the development of complications in the organs and systems of the fetus and newborn.

## 1. Introduction

The outbreak of the 2019 novel coronavirus infection disease (COVID-19) in China has attracted much attention worldwide since December 2019. The pregnant woman and her fetus are considered to be at high risk during COVID-19 infection [1]. According to the literature, the prevalence of COVID-19 among pregnant women varies, with most studies being conducted in high-income countries. In a hospital in Houston, USA, the prevalence was 8.0%. In another hospital in Connecticut, USA, the prevalence was 3.9%. In Jammu and Kashmir, India, the prevalence of COVID-19 was 3.4%, and in Zambia, it was 36.9% among pregnant women.

**Figure 1:**
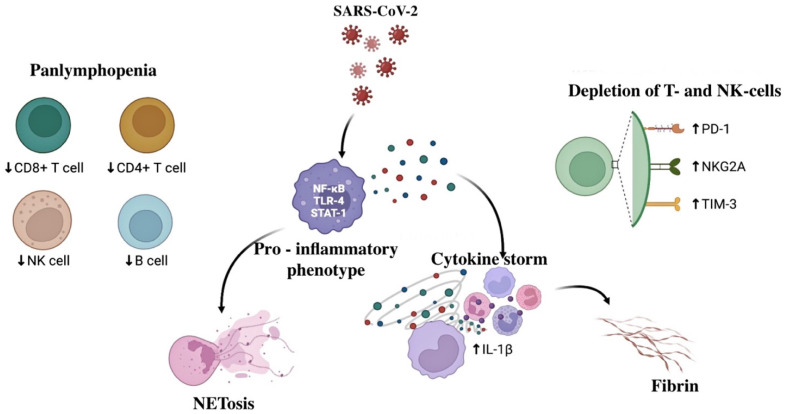
Cellular and molecular aspects of the COVID-19 pathogenesis in pregnant women (authors’ drawing). Note: PD1—programmed cell death 1; NKG2A—natural killer cell receptor; TIM-3—T cell immunoglobulin and mucin domain 3. ↑—increase in concentration. ↓—decrease in concentration.

Moreover, such figures were associated with a later date of pregnancy [2]. The outcomes of fetuses and newborns whose mothers suffered a coronavirus infection during pregnancy remain unclear. Severe COVID-19 forms develop in pregnant women with existing concomitant diseases of the lungs, cardiovascular system, kidneys, and liver, as well as the presence of arterial hypertension, diabetes mellitus, obesity, antiphospholipid syndrome (APS), and thrombophilia. COVID-19 may negatively impact the fetus through the activation of the fetal and maternal immune systems, which may be accompanied by fetal inflammatory response syndrome (FIRS). In a recent paper by Salvatore et al., who conducted a histological study of 975 placentas from women infected with COVID-19 during pregnancy, it was shown that there were no specific pathological manifestations in SARS-CoV-2 infections, even though a significant part of the placentas showed signs of inflammation, possibly related to the cytokine storm caused by the virus, without significant perinatal consequences [3]. However, according to other authors, the hyperinflammatory response, evident in SARS-CoV-2 infections compared to other coronavirus infections, can contribute to the severe course of the disease in both the mother and fetus [4,5]. Hyperinflammation is associated with neutrophil release and infiltration in various organs with the formation of neutrophil extracellular traps (NETs) and cytokine storm. Severe COVID-19 cases are characterized by an increase in pro-inflammatory cytokines plasma levels, especially interleukins IL-1β, IL-2, IL-6, IL-7, and IL-10 granulocyte-macrophage colony-stimulating factor (GM-CSF) and tumor necrosis factor-alpha (TNF-a) [6]. In addition, cytokine levels are inversely correlated with the number of CD4+ and CD8+ T cells, B cells, and natural killer (NK) cells, manifesting as lymphopenia in the peripheral blood, causing an uncontrolled hyperinflammatory response in COVID-19 [7]. Despite the decrease in the number of T cells, the immune response to COVID-19 is characterized by an increase in T helper 17 (Th17) with a simultaneous decrease in regulatory T cells (Treg), which subsequently leads to a decrease in the Treg/Th17 ratio (Figure 1). Studies have shown that the uncontrolled release of pro-inflammatory cytokines in COVID-19 cases is due to excessive activation of the Th17 immune response [5]. However, the cytokine storm immune mechanisms in COVID-19 still need to be fully understood. Interestingly, cytokine storm has also been described in several severe acute respiratory syndromes such as Middle East respiratory syndrome (MERS), severe acute respiratory syndrome (SARS), and seasonal influenza (H5N1 and H1N1). Cytokine profiles in various respiratory syndromes were similar, indicating common pathogenesis: SARS revealed elevated concentrations of IL-1, IL-6, and IL-12; transforming growth factor beta (TGF-β); interferon gamma (IFN-γ); IFN-γ inducible monokine (MIG); IL-8; MERS—IFN-α, IL-1β, IL-2, IL-6, and IL-8; and influenza H1N1—IFN-γ, TNF-α, IL-6, IL-8, IL-9, IL-17, and IL-15, and with influenza H1N5, there were increased levels of IFN-γ, IL-6, IL-8, IL-10, and MIG [8].

A recent cytokine profile study in patients with respiratory infections of various etiologies found that inflammatory cytokines, including IL-1β, IL-6, IL-8, and soluble TNF-1 receptor (sTNFR1), were significantly elevated in patients with COVID-19 compared with patients without coronavirus infection in the intensive care unit [9]. This study is consistent with a recent meta-analysis showing elevated levels of inflammatory markers such as procalcitonin, C-reactive protein (CRP), and IL-6 in patients with COVID-19 [10], and other studies that describe changes in inflammatory markers such as IL-6, IL-1β, IL-10, TNF-a, GM-CSF, and IL-17 [11,12].

**Figure 2:**
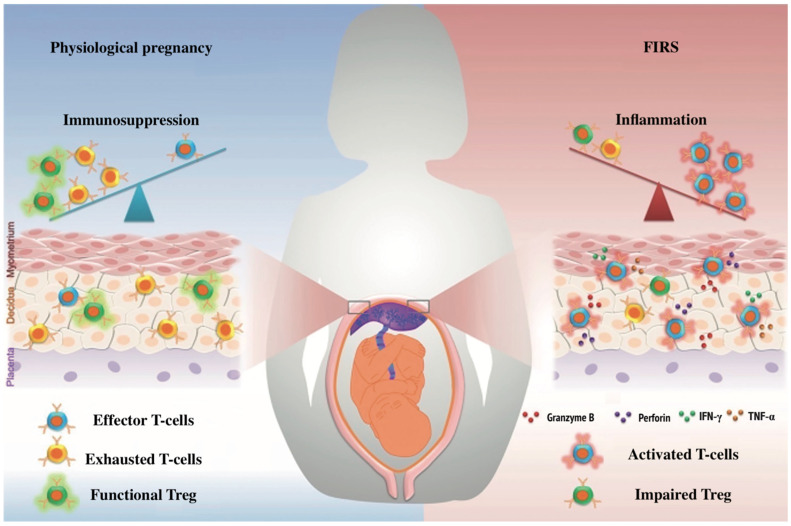
Fetal inflammatory response syndrome (FIRS). Note: IFNy—interferon-gamma; TNFa—tumor necrosis factor-alpha; Treg—T regulatory cells.

Maternal pro-inflammatory cytokine levels can cross the placental barrier and stimulate inflammatory responses and the activation of the fetus’s immune system, leading to organ damage with negative consequences for fetal development [13]. Cytokine storm can trigger acute respiratory distress syndrome (ARDS), multiple organ failure, and death. Uncontrolled systemic inflammation can cause poor pregnancy outcomes such as miscarriage, preterm birth, fetal distress, preeclampsia, intrauterine growth retardation (IUGR), and FIRS [5]. FIRS is a pathological condition characterized by the activation of the fetal immune system and increased production of pro-inflammatory cytokines, which can ultimately lead to multiple organ failure in the fetus and newborn (Figure 2). As is known, a typical clinical picture of COVID-19 is fever, which in the early stages of pregnancy, can cause congenital structural abnormalities affecting the neural tube, heart, kidneys, and other organs. However, a recent study involving 80,321 pregnant women showed that the incidence of fever in early pregnancy was 10%, while the incidence of fetal malformations in this group was 3.7%. Currently, there are no data on the risk of congenital malformations when infected with COVID-19 during the first or beginning of the second trimester of pregnancy. Nevertheless, pregnant women with COVID-19 are subject to a detailed morphological examination at 18–24 weeks of pregnancy [14]. Additionally, it is described that coronavirus infection in the mother during pregnancy leads to thymus involution in the fetus [15,16,17].

As we learn to live with COVID-19, it should be studied more deeply so that we can prevent it from having harmful effects on fetuses.

**Purpose**: Evaluation of the inflammatory response in the fetus due to the effects of COVID-19 on the mother during pregnancy by determining pro-inflammatory cytokines, cell markers, T regulatory cells, T cell response, assessment of cardiac function, and thymus size.

## 2. Materials and Methods

### 2.1. Study Design

A prospective randomized study involved 92 patients hospitalized in the SBIH Vorohobov’s City Clinical Hospital 67 MHD for delivery from May 2020 to December 2021.

#### 2.1.1. Patient Groups

The main group included 92 pregnant women with a history of coronavirus infection: SARS-CoV-2 PCR-positive pregnant women 4 weeks before delivery (subgroup 1; *n* = 30); SARS-CoV-2 PCR-positive earlier during pregnancy (subgroup 2; *n* = 32).

The control group consisted of 30 healthy pregnant women.

#### 2.1.2. Inclusion and Exclusion Criteria

Main group inclusion criteria: age > 18 years; pregnant women with a coronavirus infection during pregnancy; the presence of COVID-19 established according to molecular genetic testing (PCR) during pregnancy; singleton pregnancy; voluntary informed consent to participate in the study.

Exclusion criteria: age < 18 years; an active infectious and/or the presence of an inflammatory process; confirmed positive test for antibodies to HIV; markers of viral hepatitis, syphilis; women who had ARVI and were vaccinated; multiple pregnancies and Rh and AB0 isoimmunization; chromosomal abnormalities, genetic mutations, and congenital malformations in the fetus; refusal to participate in the study.

### 2.2. Study Methods

Pregnant women’s venous blood and their newborns’ cord blood were collected, and cell markers were analyzed. We used the multicolor flow cytometry method to determine the cell receptors’ expression level. Marker expression was assessed according to the protocol developed by the antibody kit manufacturer (Beckman Coulter, Brea, CA, USA) [18]. Anti-CD45 labeled with PacificBlue, anti-CD14 labeled with APC, anti-CD64 (FcγRI) labeled with PE, anti-CD16 (FcγRIII) labeled with PC7, anti-CD32 (FcγRII) labeled with PC5, anti-CD11b (C3b), PE-labeled, anti-CD11c (ITGAX), FITC labeled, anti-CD3-FITC (clone BW264/56), anti-CD45RO-PerCP (UCHL1), anti-CD8-PE (clone BW135/80), anti-CD4-FITC (clone M-T466), anti-CD4-APC (clone M-T466), anti-CD80-PE (clone 2A2), anti-CD86-FITC (clone 3D7), anti-CD25-APC (clone 4E3), anti-CD127-PE (clone MB15-18C9), anti-Foxp3-PE (clone 3G3), and their corresponding isotype controls to exclude autofluorescence of samples (IgG1 or IgG2). A Navios flow cytometer (Beckman Coulter, USA) was used to collect data. Analysis of the data was performed using Kaluza software (Beckman Coulter, USA). When phenotyping Treg cells, we focused not only on the CD25+ receptor molecules (the alpha chain of the IL-2 receptor) but also on the Foxp3 gene product (forkhead box p3—a transcription factor responsible for the differentiation of Treg cells, and we also determined Treg by a low expression receptor for IL-7 (CD127)) [19].

An analysis of cellular immunity was also carried out on the interferon-gamma secretion [20]. In total, 50 µL of whole blood, 150 µL of RPMI1640 medium, and a mixture of peptides (15 a.a.) completely overlapping the sequence of S and N proteins of SARS-CoV-2 were added to the wells of a 96-well round-bottom plate. It was incubated for 24 h at 37 C, 5% CO_2_; 100 μL of the supernatant was taken, and the interferon-gamma concentration was determined using flow cytometry.

In addition, the levels of circulating cytokines and the cytokine-producing ability of blood cells were analyzed. Cytokine profiles in maternal plasma and cord blood samples were analyzed with a flow immunofluorescence assay using the MILLIPLEX Map multiplex kit (Millipore, Burlington, MA, USA) according to the manufacturer’s instructions [21]. Samples were collected and analyzed on a Luminex analyzer using Bio-Plex Manager 6.0 software (Bio-Rad, Hong Kong, China). Lymphocyte cytokine-producing capacity was assessed after stimulation for 5 h with PMA (50 ng/mL) and ionomycin (2 μg/mL) in the presence of brefeldin A (10 μg/mL, Sigma-Aldrich). Cells were incubated with V450-labeled anti-IFN-gamma (B27), BD Biosciences, and PE-labeled anti-TNF-a (MAb11), BD Biosciences, and then analyzed with a Navios flow cytometer (Beckman Coulter, USA). Analysis of the data was performed using Kaluza software (Beckman Coulter, USA). Cytokines in culture supernatants were measured using an enzyme immunoassay (ELISA) per standard protocol. Optical densities were recorded at 450 nm on a microplate reader. Cytokine concentrations were calculated by extrapolating absorbance values from standard curves. Known concentrations were added on an absorbance plot using built-in software.

All newborns underwent an ultrasound examination of the thymus gland and echocardiography of the heart on the 3rd and 30th days of life. This study was conducted by the same specialist on an ultrasound machine, Logiq 500 (General Electric Medical Systems, Waukesha, WI, USA), using linear sensors with a frequency of 5–10 MHz.

### 2.3. Ethical Aspects

The study was approved by the local ethics committee of the First Moscow State Medical University (Sechenov University) of the Russian Ministry of Health, Protocol No. 04-22, dated 16 February 2022. All patients participating in the study were informed about the study’s nature and the inclusion of the results in the research work.

Informed consent was obtained from all patients to participate in the study and to process personal data by the World Medical Association’s Declaration of Helsinki (WMA Declaration of Helsinki—Ethical Principles for Medical Research Involving Human Subjects, 2013).

### 2.4. Statistical Analysis

Statistical analysis included the calculation of descriptive statistics: mean (M), median (Me), standard deviation (SD), age and body mass index, and laboratory parameters. The distribution normality assessment was carried out using the Jarque–Bera test. The data obtained were systematized in Microsoft Office Excel 2021 spreadsheets. Statistical data processing was performed using Microsoft Excel spreadsheets (Microsoft, Redmond, WA, USA) and the IBM SPSS Statistica v.13.0 software package (StatSoft Inc., St Tulsa, OK, USA). The Mann–Whitney test was used to compare quantitative data. To test the statistical significance of the factors, a one-way analysis of variance was used by calculating Fisher’s exact test, the value of which was less than 0.05, indicating the presence of a statistically significant difference; a value of Fisher’s test *p* > 0.05 indicated the absence of a difference.

## 3. Results and Discussion

### 3.1. Clinical and Anamnestic Data

The data of the clinical and anamnestic examination and perinatal outcomes are presented in Table 1. There were no significant differences in age and body mass index (BMI) among the examined patients of the main and control groups. Thus, the average age of the mothers in subgroup 1 was 34 years and BMI 28 kg/m^2^; in subgroup 2, the average age was 32 years and BMI 27 kg/m^2^; in the control group, the average age was also 34 years and BMI 25 kg/m^2^. Gestational age was less than 37 weeks in 7 out of 62 of the participants. There was no perinatal mortality in the main group and one case in the controls.

### 3.2. Analysis of Cytokine Content and Cytokine-Producing Ability of Cells

**Figure 3:**
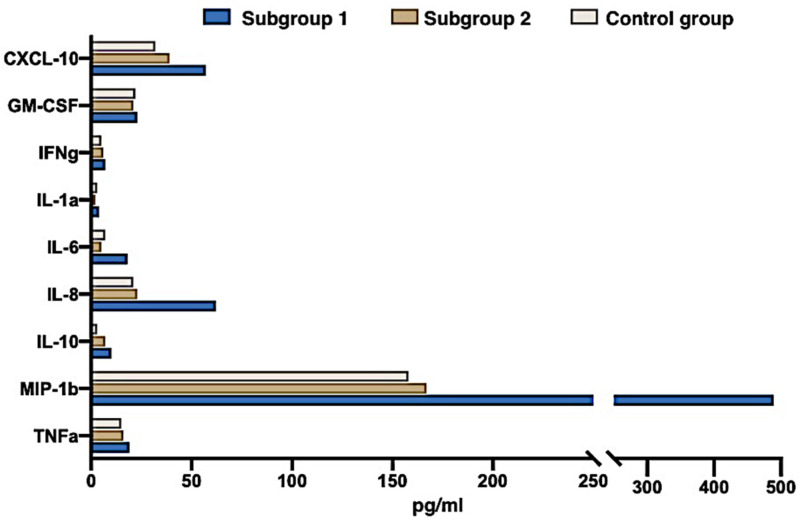
The cytokines and chemokines concentration in the cord blood of newborns from SARS-COV-2-positive mothers 4 weeks before delivery (group 1), SARS-CoV-2-positive mothers earlier during pregnancy (group 2), and the control group (group 3).

Table 2 presents a comparative analysis of cytokine content in the umbilical cord blood. Significantly higher levels of pro-inflammatory cytokines, in particular, IL-1a, IL-6, IL-8, IL-10, TNF-a, and chemokines such as chemokine-10 (C-X-C motif chemokine ligand 10, CXCL), were detected in the examined newborns from subgroups 1–10 and inflammatory macrophage protein-1β (macrophage inflammatory protein-1β, MIP-1β) compared with the control group (Figure 3). The content of cytokines in peripheral blood in the newborns of subgroup 2 and the control group did not differ significantly. At the same time, the content of cytokines in the peripheral blood of the mothers of the main group did not significantly differ from their concentrations in the mothers of the control group at the time of delivery.

### 3.3. Determination of Cellular Markers

**Figure 4:**
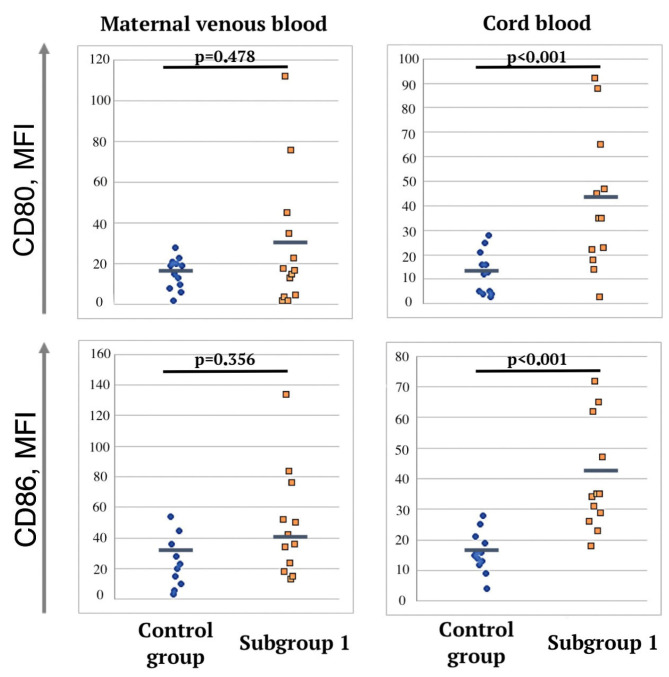
Markers of dendritic cell activation in cord and peripheral blood samples of pregnant women in control group and subgroup 1.

We analyzed the expression of CD80 and CD86 activation markers on dendritic cells. In newborns from the mothers of subgroup 1, a significant increase in CD80 and CD86 expression was detected in umbilical cord blood compared to the control group (Figure 4). In the newborns of subgroup 2, no significant differences were found with the control group. Thus, the median fluorescence intensity (MFI) of the activation marker of CD80 dendritic cells in subgroup 1 was 45.2 (35; 65.1), and in newborns from the mothers of the control group, it was 13.8 (4.9; 19.8) (*p* < 0.001, U-test). The MFI of the CD86 activation marker in subgroup 1 was 41.8 (36.8; 69.4), and in the control group, it was 17.8 (6.9; 25.1) (*p* < 0.001, U-test).

**Figure 5:**
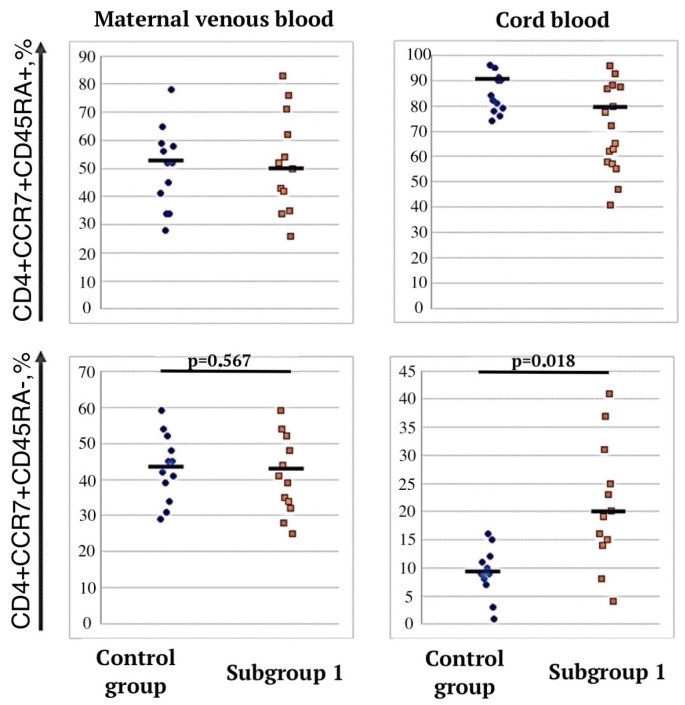
Central memory T cells in cord and peripheral blood samples of pregnant women in control group and subgroup 1.

In the umbilical cord blood samples of subgroup 1, the proportion of CD4+CCR7+CD45RA T cells increased with a concomitant decrease in the proportion of naive CD4+CCR7+CD45RA+ T cells compared with the control group (*p* = 0.018)—21.0% (14; 28) vs. 9% (2; 13) (Figure 5), and in the newborns of subgroup 2, there were no significant differences from the control group.

### 3.4. Determination of the Functional Activity of Treg Cells

**Figure 6:**
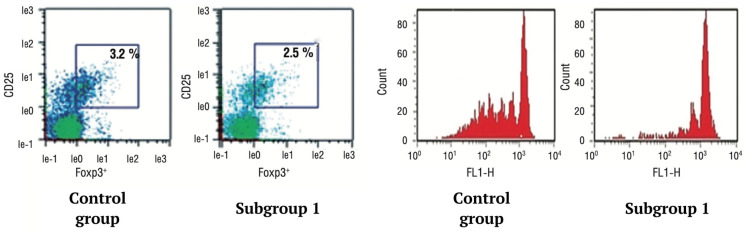
Regulatory T cell quantitative and functional characterization.

No significant differences between the groups were found when studying the quantitative and functional characteristics of Treg cells in mothers. However, in newborns, it was shown that in subgroup 1, the functional activity of Treg was significantly suppressed. The suppression coefficient of cell proliferation (%) was 43.8 (29.4; 58.6) versus 71.2 (53.1; 78.6) (*p* < 0.001) compared with the control group (Figure 6).

**Figure 7:**
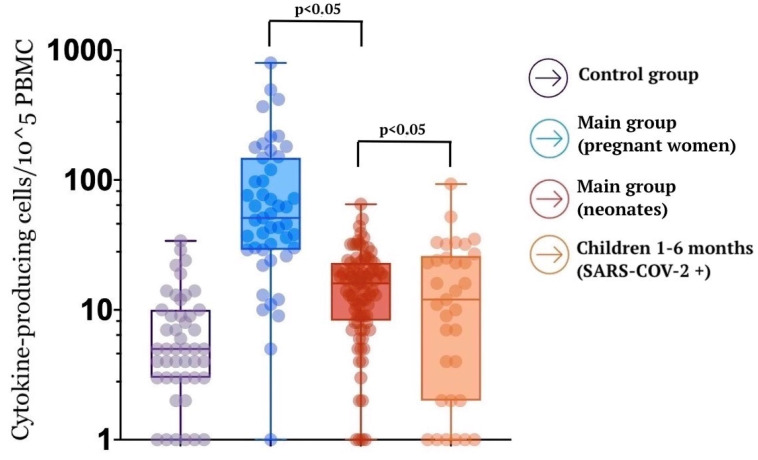
SARS-CoV-2-specific T cell memory magnitude in study groups.

A specific T cell memory study found that pregnant women demonstrate a strong SARS-CoV-2-specific T cell response (Figure 7).

**Figure 8:**
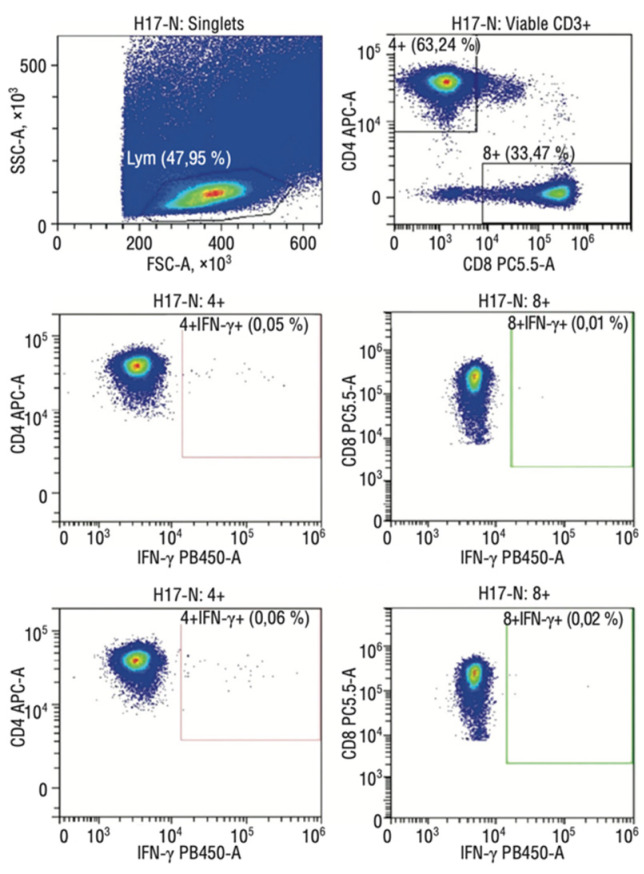
Flow cytometry imaging of fetal SARS-CoV-2-specific T cell subsets in mild COVID-19 convalescent mothers.

**Figure 9:**
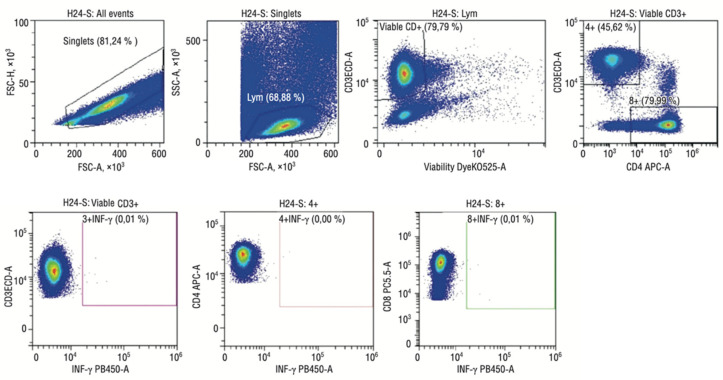
Flow cytometry imaging of fetal SARS-CoV-2-specific T cell subsets in severe COVID-19 convalescent mothers.

At the same time, specific memory T cells were also detected in 32% of the newborns’ cord blood samples, which does not exclude the possibility of intrauterine infection transmission. Moreover, the specific T cell response was higher in newborns whose mothers had a coronavirus infection during pregnancy than in children who became ill in the postnatal period (the first six months of life). Of interest is that all pregnant women with severe COVID-19 showed a weak specific T cell response, and in their newborns, memory cells were not detected in their cord blood samples. It can be assumed that maternal cells can accelerate the fetal immune cells’ maturation by providing essential growth and differentiation factors (Figure 8 and Figure 9).

**Figure 10:**
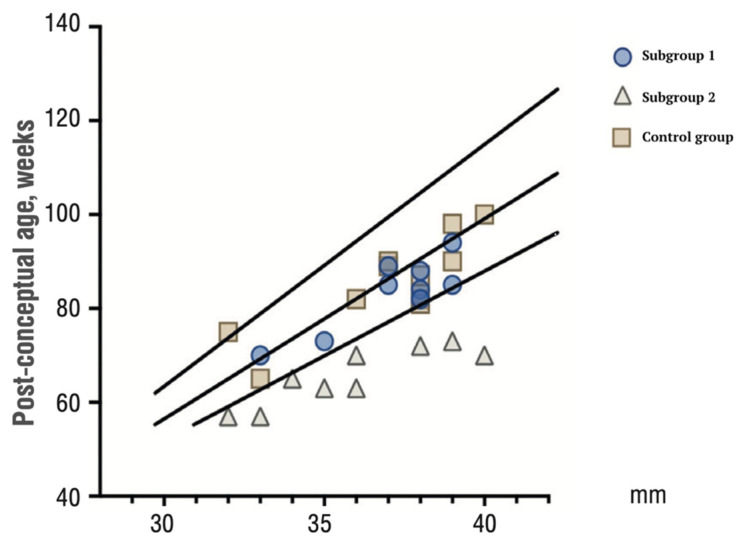
Centile plot of thymus gland size in neonates from paired SARS-CoV-2-positive mothers 4–6 weeks before delivery (subgroup 1), SARS-CoV-2-positive mothers earlier during pregnancy (subgroup 2), and the control group.

An increase in maternal pro-inflammatory cytokines that activate the fetal immune system has become known as FIRS. At the same time, the factors responsible for the manifestation of FIRS in the form of multiple organ failure in the fetus and newborns are not currently reliably identified, although changes in the fetal thymus are known. The rationale for this study is the evaluation of the fetal thymus involution. Following this situation, we found thymus involution in newborns against the background of COVID-19 in the mother (Figure 10).

**Figure 11:**
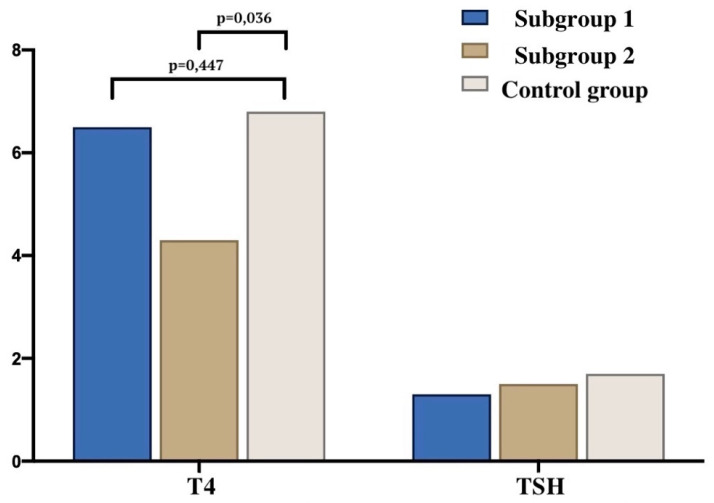
The concentration of T4 and TSH in newborns and mothers in the study groups.

Our results are consistent with data from the literature in terms of how the fetal thymus actively responds to systemic maternal inflammation during the gestational period [16,22,23]. Hyperinflammation caused by high circulating pro-inflammatory cytokines and chemokines in severe COVID-19 may impair the placental barrier and promote intrauterine SARS-CoV-2 infection transmission [24,25,26]. It is also suggested that intrauterine vertical transmission to the fetus does occur, albeit rarely. Therefore, there is a significant possibility of SARS-CoV-2 having an impact on newborns. Whether this is an immune-mediated event or a direct cytopathic effect of the virus requires further investigation [27,28]. We found a decrease in the free thyroxine (T4 light) concentration in newborns from mothers who had a coronavirus infection during pregnancy (subgroup 2) compared with a healthy control group (*p* = 0.036) (Figure 11). The observed transient hypothyroxinemia in these newborns may be due to the immaturity of the hypothalamic–pituitary–thyroid system [29], the syndrome of euthyroid pathology (non-thyroidal illness) [30]. Recent studies have shown an association between reduced T4 levels in preterm infants with impaired cognitive and motor development [31]. Thus, it can be assumed that COVID-19, in the long term, affects not only the thymus but also the thyroid gland.

Table 3 presents the echocardiographic parameters of newborns whose mothers suffered a COVID-19 infection during pregnancy and from healthy mothers on days 3 and 30 of their newborns’ lives. In newborns from mothers who had SARS-CoV-2, the indicators were significantly different from the control group. Changes in functional parameters indicate remodeling of the heart chamber and a possible limitation of the reserve capacity of myocardial contractile activity.

It is known that SARS-CoV-2 penetration and viral replication in cells occur through binding to human angiotensin-converting enzyme II (hACE-2) receptors, primarily observed in cardiac tissue, explaining complications such as myocarditis and myocardial dysfunction [32]. It is also believed that myocardial injury results from direct and indirect damage to cardiomyocytes caused by a systemic inflammatory response, leading to myocarditis, decreased systolic function, and tachyarrhythmia [33]. Our study found that hyperinflammation caused by SARS-CoV-2 also leads to functional changes in the myocardium of newborns from mothers who had COVID-19.

It is important to note that cytokines such as TNF-a, IL-1, IFNγ, IL-4, and IL-10, as well as the overproduction of chemokines such as CXCL10 and CXCL11, in severe cases, are associated with increased incidence of adverse outcomes [34], and IL-6 excessive production is associated with adverse pregnancy outcomes such as preterm birth, premature membranes rupture, and chorioamnionitis [35].

In addition, elevations in IL-6 levels have also been observed in severe cases of COVID-19 and are seen as one of the key players in the cytokine storm [34,36]. It is also known that an increase in CXCL10 indicates an unusual inflammatory response that manifests itself in graft-versus-host disease [37]. Our results show that significantly higher IL-6 and CXCL10 levels were found in the main group, which requires long-term follow-up of these patients. Further study of this problem is needed to find a suitable treatment and the prevention of the hyperinflammatory process during pregnancy.

Recent studies have suggested a critical T cell role in the clearance of SARS-CoV-2 and protection against severe COVID-19 development. In particular, the coordination of adaptive immune responses, including those of CD4+ T cells, CD8+ T cells, and antibodies, is required to fight COVID-19. Additionally, peak disease severity is inversely correlated with the incidence of SARS-CoV-2-specific CD4+ and CD8+ T cells instead of SARS-CoV-2 antibody titers [38]. Other studies found a T cell response in recovering COVID-19 patients with anti-SARS-CoV-2 IgG [39]. Specific memory T cell retention in post-COVID-19 patients ten months after infection has been shown regardless of disease severity [40]. To summarize, these studies powerfully demonstrate the protective role of T cells in COVID-19. Our study also identified a SARS-CoV-2-specific T cell response in pregnant women and their fetuses.

The main strength of our study was that we studied the deregulation of the cellular regulatory link of the fetal immune system at the level of Treg lymphocytes, conducted a comparative analysis of the cytokine profile of the mother and newborn after a coronavirus infection, as well as the level of expression of cellular markers. The limitation of our study is that we do not know how long the activation of the immune system persists after birth due to a limitation in terms of newborn supply.

## 4. Conclusions

Our results show that COVID-19 negatively affected the fetal immune system. However, disease severity in a pregnant woman does not correlate with the severity of systemic inflammatory response syndrome in the neonatal period. It can vary from minimal changes in laboratory parameters to sepsis and septic shock development. Pregnant women diagnosed with COVID-19 should be treated as high-risk patients, accompanied by qualified professionals, frequent follow-up examinations, and adequate laboratory testing. Thus, in clinical practice, the best treatment these infants can receive is to monitor them both in utero and in the long term to provide appropriate and personalized medical care.

## Figures and Tables

**Table 1:** Clinical and anamnestic characteristics and perinatal outcomes.

	Main Group		
Characteristic	Subgroup 1 *n* = 30	Subgroup 2 *n* = 32	Control Group *n* = 30
Age (years Me ± SD)	34.0 ± 4.1	32.3 ± 3.2	34.0 ± 3.7
Body mass index, Me ± SD	28 ± 2.8	27 ± 2.2	25 ± 2.5
Severe COVID-19, *n*	7	6	–
Gestational age < 37 weeks, *n*	3	4	1
Apgar score at the 5th minute Me ± SD	7 ± 0.7	8 ± 0.5	9 ± 0.3

**Table 2:** Cytokines and chemokines level in umbilical cord blood of newborns.

Parameter	Control Group *n* = 30		Subgroup 1 *n* = 30		*p* U-Test
	Me	Q_1_–Q_3_	Me	Q_1_–Q_3_	
GM-CSF	19.2	14.6–28.7	23.3	15.4–47.1	0.582
IFN-γ	4.4	3.5–6.9	6.3	3.2–11.9	0.436
IL-1α	1.8	1.2–5.3	6.5	1.8–21.3	0.018
IL-6	2.9	1.3–10.1	13.8	6.1–38.4	0.032
IL-8	21.9	6.7–116.0	52.7	23.1–418.3	0.061
IL-10	5.4	2.1–13.0	12.5	7.7–49.1	0.003
MIP-1β	154.8	136.1–287.7	501.2	247.6–1648.1	<0.001
TNF-α	12.1	9.1–18.3	25.2	16.9–28.7	<0.001
CXCL-10	25.1	9.8–32.1	68.4	37.1–92.6	<0.001

Note: GM-CSF—granulocyte-macrophage colony-stimulating factor; IFN-γ—interferon-gamma; IL—interleukin; MIP-1β—macrophage inflammatory protein-1β; TNF-α—tumor necrosis factor-alpha; CXCL 10—chemokine (C-X-C motif) ligand 10; significant differences are highlighted.

**Table 3:** Main echocardiography parameters in newborns from mothers with COVID-19 during pregnancy.

Indicator (Me)	Main Group		Control Group		*p*
	3 Days	30 Days	3 Days	30 Days	
**ESR, cm**	0.82	0.86	0.91	1.37	<0.0001
**ESD, mL**	1.28	1.45	1.72	4.35	<0.0005
**EDR, cm**	1.31	1.44	1.6	1.96	<0.001
**EDV, mL**	4.41	5.36	5.8	11.9	<0.0001
**SV, mL**	3.17	3.82	4.26	7.9	<0.0001

Note: ESR—end-systolic dimension; ESD—end-systolic volume; EDR—end-diastolic dimension; EDV—end-diastolic volume; SV—stroke volume.

## Data Availability

The data are available at department Obstetric gynecology perinatology Sechenov University and can be requested if meta-analysis can be done.
